# 3D Surface Reconstruction of Plant Seeds by Volume Carving: Performance and Accuracies

**DOI:** 10.3389/fpls.2016.00745

**Published:** 2016-06-07

**Authors:** Johanna Roussel, Felix Geiger, Andreas Fischbach, Siegfried Jahnke, Hanno Scharr

**Affiliations:** Institute of Bio- and Geo-sciences, IBG-2: Plant Sciences, Forschungszentrum Jülich GmbHJülich, Germany

**Keywords:** automated seed handling, *Arabidopsis*, image processing, silhouette, performance analysis

## Abstract

We describe a method for 3D reconstruction of plant seed surfaces, focusing on small seeds with diameters as small as 200 μm. The method considers robotized systems allowing single seed handling in order to rotate a single seed in front of a camera. Even though such systems feature high position repeatability, at sub-millimeter object scales, camera pose variations have to be compensated. We do this by robustly estimating the tool center point from each acquired image. 3D reconstruction can then be performed by a simple shape-from-silhouette approach. In experiments we investigate runtimes, theoretically achievable accuracy, experimentally achieved accuracy, and show as a proof of principle that the proposed method is well sufficient for 3D seed phenotyping purposes.

## 1. Introduction

Making image analysis methods available for plant phenotyping applications is currently a driving force in plant sciences (Spalding and Miller, 2013). In many such applications the absence of suitable image processing is even a bottleneck (Minervini et al., 2015). More than 100 specialized methods (Lobet et al., 2013) and software packages are available for image-based analysis of different plant parts, e.g., fruit shape (Brewer et al., 2006), single or multiple leaves (Bylesjö et al., 2008; Weight et al., 2008; Alenya et al., 2011; De Vylder et al., 2011; Wallenberg et al., 2011; Dellen et al., 2015; Müller-Linow et al., 2015; Pape and Klukas, 2015), hypocotyl and seedlings (Koenderink et al., 2009; Wang et al., 2009; Silva et al., 2013; Golbach et al., 2015), shoot (Augustin et al., 2015; Santos and Rodrigues, 2015; Pound et al., 2016), rosettes (Arvidsson et al., 2011; Aksoy et al., 2015) and many more. Such analysis tools are needed in robotic imaging platforms for high-throughput plant phenotyping (Granier et al., 2006; Jansen et al., 2009; Hartmann et al., 2011; Nagel et al., 2012; van der Heijden et al., 2012; Fahlgren et al., 2015), but also in affordable systems (Tsaftaris and Noutsos, 2009; Minervini et al., 2014; Santos and Rodrigues, 2015).

Plant seed phenotyping is needed by seed banks for quality management e.g., concerning breeding purposes, linking to germination rate or plant growth. For this, 2D scanning is a popular, affordable technique (Herridge et al., 2011; Tanabata et al., 2012; Moore et al., 2013; Whan et al., 2014). Several commercial software packages are available for seed investigations using flat-bed scanners (e.g., Regent Instruments, 2000; Next Instruments, 2015). It has been applied to different seed types, like *Arabidopsis*, soybean, barley, or rice. Typically parameters like width, length, or area are calculated from the 2D images, but also more complex shape measures like Fourier descriptors (Iwata and Ukai, 2002; Iwata et al., 2010).

However, to the best of our knowledge, no affordable 3D imaging technique has been presented so far designed for seed measurements. Correspondence-based techniques (Quan et al., 2006; Paproki et al., 2012; Pound et al., 2014, 2016; Santos and Rodrigues, 2015) reconstructing 3D models from multiple images, or other low-cost techniques like laser scanning or the Kinect can be used for 3D whole plant reconstruction (Paulus et al., 2014) or root systems in transparent gel (Fang et al., 2009). However, such techniques are not suitable for much smaller objects like seeds of rapeseed plants (~2 mm diameter) or even *Arabidopsis* seeds (~0.2–0.4 mm length).

**Figure 1:**
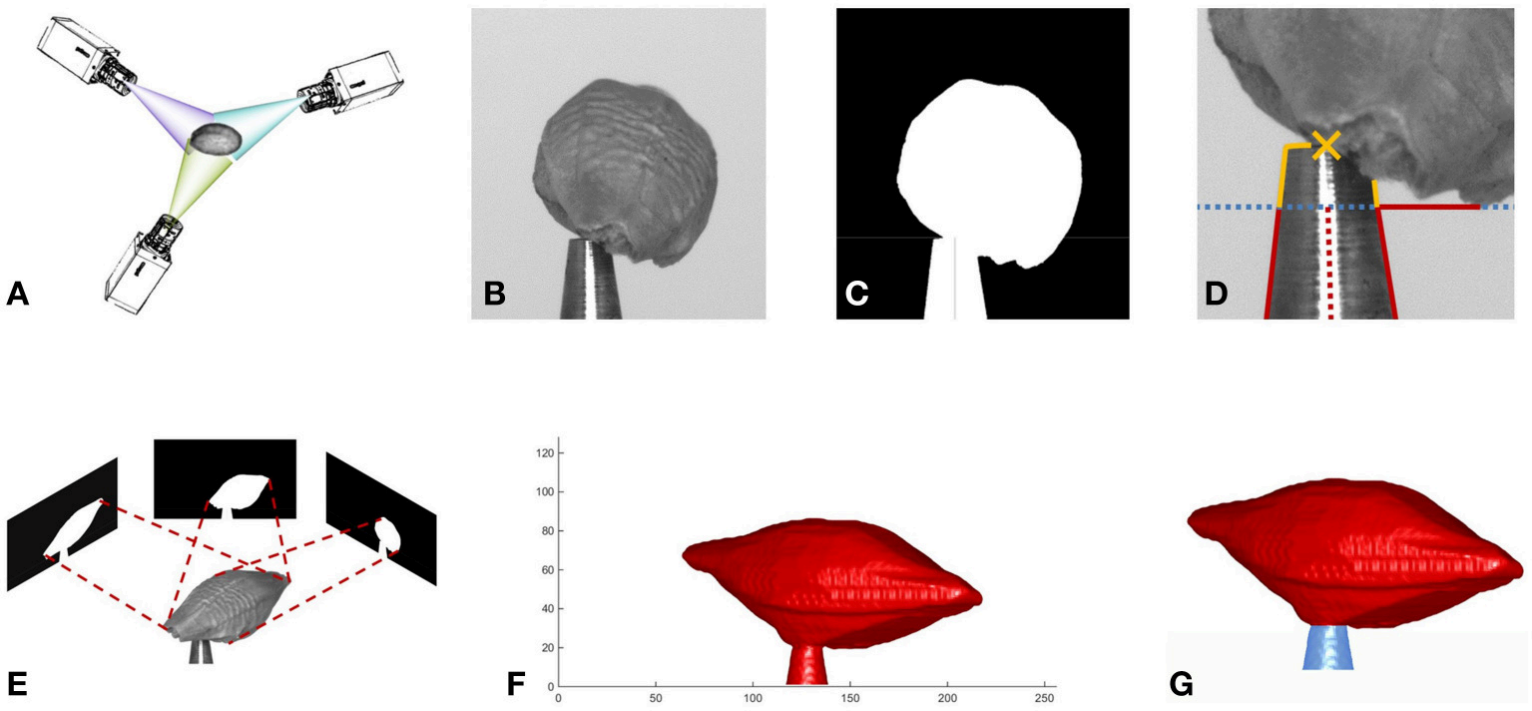
**Overview of the reconstruction method**. **(A)** Image acquisition from multiple viewing angles. **(B)** One of the acquired gray value images. **(C)** Mask image. **(D)** Estimation of tool center point (TCP). **(E)** Estimate shape from silhouette by volume carving. **(F)** Surface of reconstructed volume. **(G)** Tool removed from volume: seed red, tool blue.

Here we investigate volume carving, a well-known shape-from-silhouette technique (Martin and Aggarwal, 1983; Potmesil, 1987; Laurentini, 1994), for 3D seed shape reconstruction. It is a fast, reliable, and simple but robust method, having been used in plant phenotyping before, e.g., 3D seedling reconstruction (Koenderink et al., 2009; Golbach et al., 2015) or root system investigations (Clark et al., 2011; Zheng et al., 2011; Topp et al., 2013). Depending on the selected viewpoints it approximates the convex hull of an object or reconstructs even valleys and saddle-points, but cannot reconstruct true concavities. Most seeds are, however, relatively smooth, convex objects. For the seed types investigated here (*Arabidopsis*, barley, and maize, see Figure 1), true concavities seem to be of low relevance for volume estimation. For non-smooth seeds, like e.g., seeds of the plant parasites *Phelipanche aegyptiaca*, or *Orobanche cernua* the proposed method may be less suitable.

This paper is an extension of our conference publication (Roussel et al., 2015), thus theory (Section 2 and 3), and some experiments from Section 4 are mainly repeated from there. We extend the theory by an accuracy check and iterative camera position correction procedure, and the experiments by a numerical and experimental investigation of achievable accuracy vs. number of images in Sections 4.2 and 4.3. Further we updated references and discussion.

## 2. Reconstructing seed shape from silhouettes

Aiming at relatively simple, mostly convex seed shapes, target voxel resolutions needed to describe such shapes are comparably low—as we will show in the experiments below, see Section 4. Therefore, for this study, it is sufficient to apply one of the most basic volume carving approaches.

We get the intrinsic camera matrix **K** (Hartley and Zisserman, 2004) and the distance between the origin of our working volume and the camera center from calibration (*cmp*. Section 3.2). The origin of the working volume is selected to be the tool center point (TCP) of the robot system handling the seeds, i.e., rotating them in front of the camera for imaging (*cmp*. Section 3.1).

We acquire *N* images, showing a seed under (equidistantly spaced) rotation angles α_*i*_ where *i* ∈ {1, …, *N*}, see Figure 1. Rotation is around the vertical axis through the TCP, being parallel to the *y*-axis of the camera. We segment by gray-value thresholding each image into a binary mask **M**_*i*_ being one at the foreground, i.e., seed and tool tip, and zero at background locations. Small objects like noise are removed and small holes (e.g., the reflection of the tool) filled.

For each image and thus segmentation mask we calculate the homogeneous camera projection matrix **P**_*i*_, from the rotation angle α_*i*_ by

(1) $$\begin{array}{l} {\text{P}}_{i}=\text{K}\left({\text{R}}_{i}|{\vec{t}}_{i}\right) \end{array}$$

where **R**_*i*_ is the rotation matrix corresponding to the given angle α_*i*_, and translation vector ${\vec{t}}_{i}$ is calculated using the distance of the world origin to the camera center, also known from calibration (see e.g., Hartley and Zisserman, 2004). By this, the world coordinate frame rotates with the object, i.e., the seed.

We define an equidistantly spaced, cubic voxel grid around the world origin, being large enough to contain the seed. The thus defined working volume depends on the seed type. For *Arabidopsis* we use (1 mm)^3^, for rapeseed (2.9 mm)^3^, and for barley and maize (13 mm)^3^.

Each voxel center with homogeneous world coordinates $\vec{X}$ is projected to a point ${\vec{x}}_{i}$ in each mask **M**_*i*_ by

(2) $$\begin{array}{l} {\vec{x}}_{i}={\text{P}}_{i}\vec{X} \end{array}$$

If $\vec{X}$ is projected to the background region of at least one of the *N* masks **M**_*i*_, then this voxel does not belong to the foreground object and its value $\text{V}\left(\vec{X}\right)$ is set to 0, i.e.,

(3) $$\begin{array}{l} \text{V}\left(\vec{X}\right)=\overset{N}{\underset{i=1}{\prod }}{\text{M}}_{i}\left({\vec{x}}_{i}\right) \end{array}$$

Thus, if a voxel belongs to the foreground object, its value $\text{V}\left(\vec{X}\right)$ is set to 1.

When higher voxel resolution is desired, and thus runtimes increase, parallelization of the carving algorithm (Brenscheidt, 2014) is feasible (see Section 4.1). Even higher resolutions become available on current desktop computer hardware, when hierarchically representing the voxel grid, e.g., as an octree (Szeliski, 1993; Klodt and Cremers, 2015).

One of the main drawbacks of this simple carving algorithm is its sensitivity to imprecise external camera calibration. When a mask **M**_*i*_ is misaligned and thus does not well overlap with the “true” object volume, the non-overlapping parts are deleted from the volume without further testing or corrections. We therefore apply an image-based camera pose calibration step, as described next.

### 2.1. Correcting camera pose

Methods not adapting camera pose by estimating extrinsic parameters from the acquired images are known to be particularly sensitive to (extrinsic) calibration errors, thereby requiring precise positioning of the cameras (see e.g., Yezzi and Soatto, 2003). For relatively large objects in the multiple centimeter-range, say 20 cm long and filling most of an image, and typical pixel resolutions, say 2000 × 2000, a pixel covers an object area 0.1 × 0.1 mm^2^. Position repeatability of industrial-grade robotic systems, typically ≤ 20 μm and ≤ 0.05° (Denso Robotics Europe, 2015), is therefore high enough for precise reconstruction. However, for objects being few millimeter in size or even in the sub-millimeter range additional care has to be taken. The mathematical TCP coordinates known to the robot control software may not coincide precisely with the physical TCP at the tool tip, due to mechanical calibration inaccuracies, wear and tear, or small deformations of the tool. In our case, instead of being at a fixed location in the camera images, the TCP moves on a more or less reproducible, elliptic trajectory of up to 200 μm diameter, varying with room temperature.

**Figure 2:**
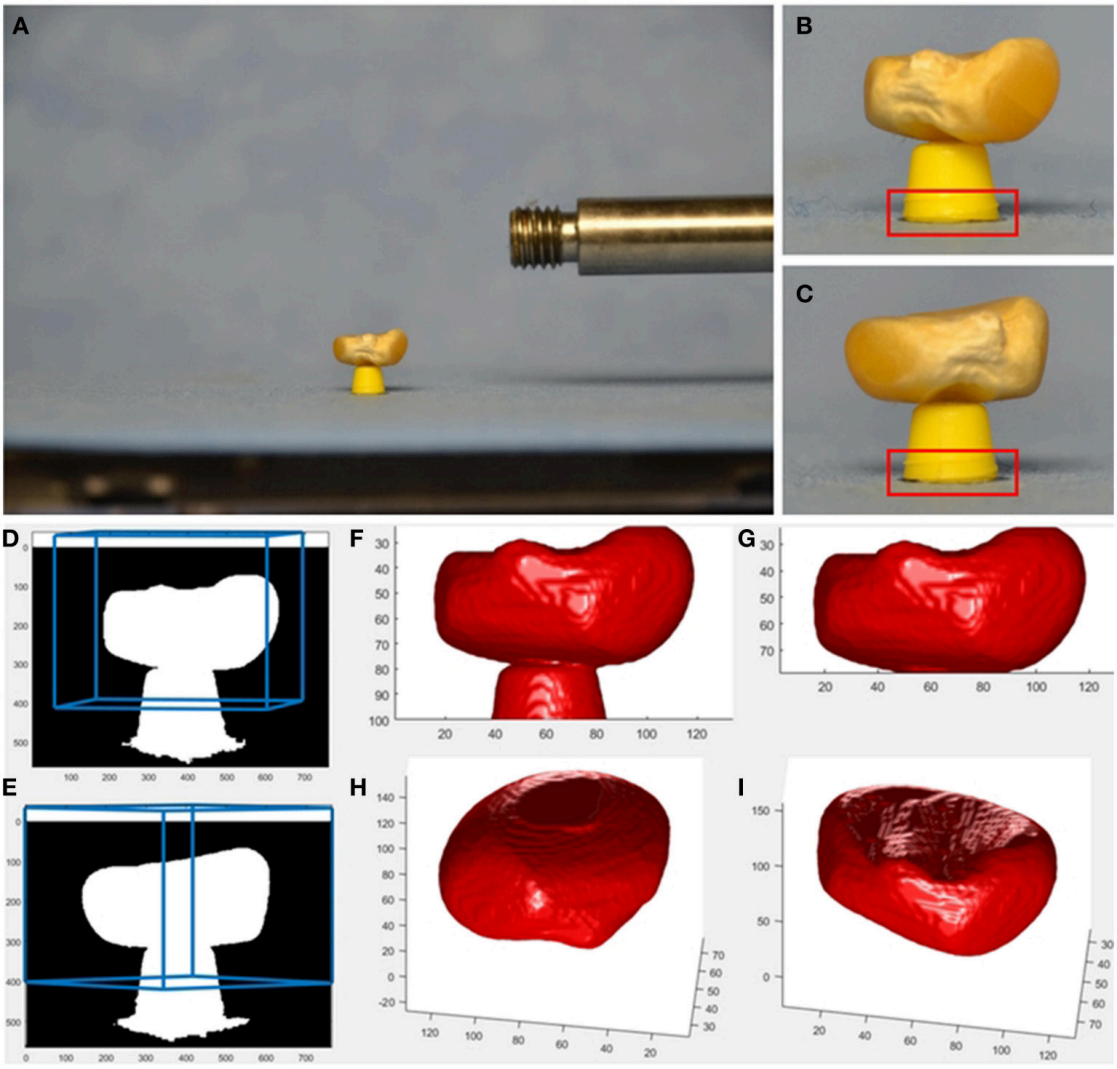
**Example provided together with source code as supplemental material**. Imaging is done using affordable hardware, i.e., a usual SLR camera (Nikon D7000, AF-S Nikkor 16–85 mm 1:3.5–5.6 GED lens at *f* = 85 mm) and a motorized turntable (Steinmeyer DT130-360°-SM01) for rotating the seed. As tool keeping the seed above the turntable's plane, we use a cut-off ball-pen-tip. A smaller tool, like in our robotic setup, allows for better seed visibility and reconstruction, however it is not easy to build. Even though the SLR is mounted on a sturdy tripod and released by a remote control, the camera center moves from image to image. In this setup, the base of the tool is visible in each image and can be used as target for correlation-based image registration. From the calculated image shifts, projection matrices **P**_*i*_ are adapted accordingly. See provided source code for more details. **(A)** SLR image showing a maize seed, **(B,C)** cropped 760×564-image with 293×100 target for cross correlation indicated in red, **(D,E)** mask image with borders of the user-defined reconstruction volume projected back into the image in blue, **(F)** reconstructed seed and tool, **(G)** reconstructed seed with tool removed, **(H,I)**, reconstructed seed from a more bottom and more top view.

Before projecting the voxels to the mask images, we therefore adapt projection matrices **P**_*i*_. If a suitable non-changing target moving with the TCP is visible in all images, image registration can be done using simple normalized cross-correlation (see Figure 2).

**Figure 3:**
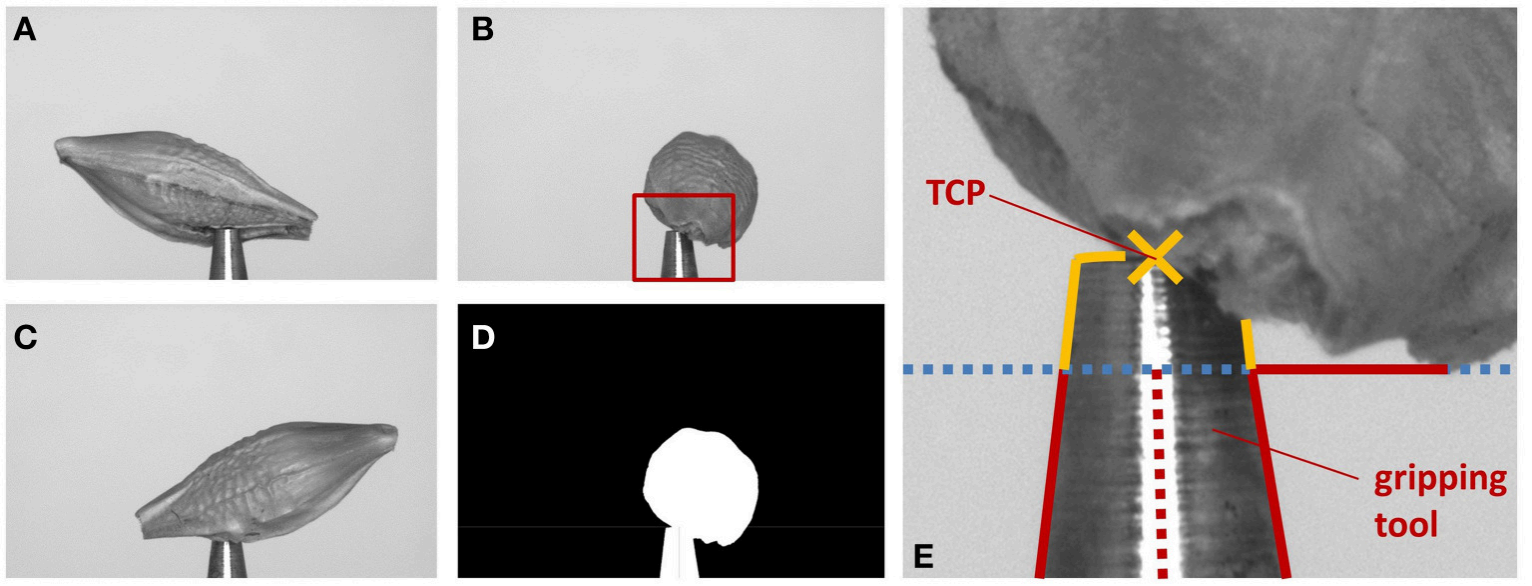
**Illustration of the extrinsic camera calibration correction**. **(A–C)** Images of the same barley seed taken from different angles. **(D)** Mask image generated from **(B)**. **(E)** Steps to find the TCP: (1) find edges of gripping tool (red lines), stop when lines diverge (blue dotted line). (2) *x*_TCP_ is average of middle between found edge positions (red dotted line). (3) Trace edges further as long as they come closer to *x*_TCP_ (yellow lines). (4) Top most position is *y*_TCP_. The found TCP is indicated by a yellow cross.

In our robotic application, in order to adapt projection matrices **P**_*i*_, the truncated cone shape of the gripping tool has to be found, see Figure 3. As larger seeds may partly occlude the tool tip, we search for a region of the tool being reliably visible in the images. The tool enters the image vertically from below and becomes smaller in diameter toward the true TCP, being the center point of the very tip of the tool. As we can robustly find the tool's left and right edges, we apply a simple and very fast procedure. We calculate the visible width of the tool line by line starting at the bottom of the image, moving upwards, i.e., in negative *y*-direction. We iterate while the width decreases and is larger than the minimum tool width (being at the tip). The thus reached *y*-coordinate is taken as first estimate of the TCP *y*-coordinate *y*_TCP_. A reliable estimate of the TCP's *x*-coordinate *x*_TCP_ is established as the mean of all found left and right edge *x*-positions. As the tool tip may be partly occluded by the seed, *y*_TCP_ needs refinement. For this the left and right tool edges are independently tracked further until the narrowest point is reached, i.e., the rightmost point of the left edge, and the leftmost point of the right edge. The smallest *y*-value (highest point) of the two points is taken as new *y*_TCP_.

For small seeds like *Arabidopsis* this procedure works reliably, as the seeds are too small to occlude the whole tool tip in an image. For larger seeds, we use the observation that the TCP's elliptic trajectory results in its *y*-coordinates to describe a sinusoidal curve over the rotation angle. We therefore robustly fit a sin-curve to the *y*-coordinates and correct outliers according to the fit result.

For such small objects, the optical lens setup (*cmp*. Section 3) features a narrow opening angle (i.e., large zoom), like a microscope at 1-to-1 magnification. This means lines of sight are almost parallel and thus depth effects are negligible. This allows to update **P**_*i*_ with ${\vec{x}}_{\text{TCP}}$ by simply setting the principal point (Hartley and Zisserman, 2004) to ${\vec{x}}_{\text{TCP}}$.

In our experiments we observed that ${\vec{x}}_{\text{TCP}}$ can be estimated reliably with pixel accuracy, when no disturbances like small dust particles are present. Maximum offset in locating TCP from an unoccluded tool tip was 2 pixel.

In situations, where larger inaccuracies in locating ${\vec{x}}_{\text{TCP}}$ occur, testing consistency of results and correcting ${\vec{x}}_{\text{TCP}}$ is recommended. Back-projection of the reconstructed 3d object is a simple procedure allowing to test whether or not **P**_*i*_ is correct and the selected segmentation procedure is suitable. For this test, each surface voxel of the found 3d object is projected to a mask image ${\tilde{\text{M}}}_{i}$, initially filled with zeros. A voxel is projected to ${\tilde{\text{M}}}_{i}$ by projecting its corners to ${\tilde{\text{M}}}_{i}$ using **P**_*i*_ and filling the respective convex hull with ones. If no errors occurred, the thus generated foreground mask should be identical to the segmentation mask **M**_*i*_ (up to ignored subpixel effects when filling the convex hull, leading to a potentially slightly dilated mask ${\tilde{\text{M}}}_{i}$). Measuring overlap between the two masks can be done using well established measures, e.g., the *Overlap Ratio Criterion* $=|{\tilde{\text{M}}}_{i}\cap {\text{M}}_{i}|∕|{\tilde{\text{M}}}_{i}\cup {\text{M}}_{i}|$ (see e.g., Everingham et al., 2010), or *Dice Similarity Coefficient* $=2|{\tilde{\text{M}}}_{i}\cap {\text{M}}_{i}|∕\left(\left|{\tilde{\text{M}}}_{i}|+|{\text{M}}_{i}\right|\right)$ established by Dice (1945) and Sørensen (1948), where | · | denotes set cardinality. These measures are used throughout image analysis and are common in plant imaging as well (Minervini et al., 2014).

In case the achieved mask overlap is too small but larger than zero, iterative procedures can be applied to increase accuracy. Straight forward is to (1) shift the principal point in each projection matrix **P**_*i*_ such that the center of mass of ${\tilde{\text{M}}}_{i}$ coincides with the center of mass of **M**_*i*_ and (2) recarve, and iterate both steps until convergence or suitably large overlap. Alternatively, gradient descent-based algorithms optimizing camera pose may be applied as a refinement step (e.g., Yezzi and Soatto, 2003).

### 2.2. Removing the tool from the seed

For small seeds not overlapping with the tool, the TCP lies precisely in the world origin, i.e., the origin of the reconstructed voxel block. Thus, voxel above the TCP contain the seed, voxel below (which in that case we do not reconstruct) contain the tool. In cases where seed and tool may overlap (see e.g., Figure 3), the tool tip is also reconstructed. It can be removed from the volume data using its known position, orientation, and physical size by deleting the corresponding voxel volume.

Alternatively, at high voxel resolutions, where the reconstructed volume covered by the tool may be affected by noise, one can estimate the tool position from the reconstruction. Summing up voxel values of horizontal planes in the bottom region of the volume gives reliable estimates of the area of horizontal cuts through the tool. While the areas decrease when summing over higher and higher planes, the planes are deleted from the data. Then, when areas no longer decrease, using these areas, we estimate the *y*-position of the truncated cone using a least squares fit and remove the thus covered volume.

## 3. Materials and methods

### 3.1. Imaging

**Figure 4:**
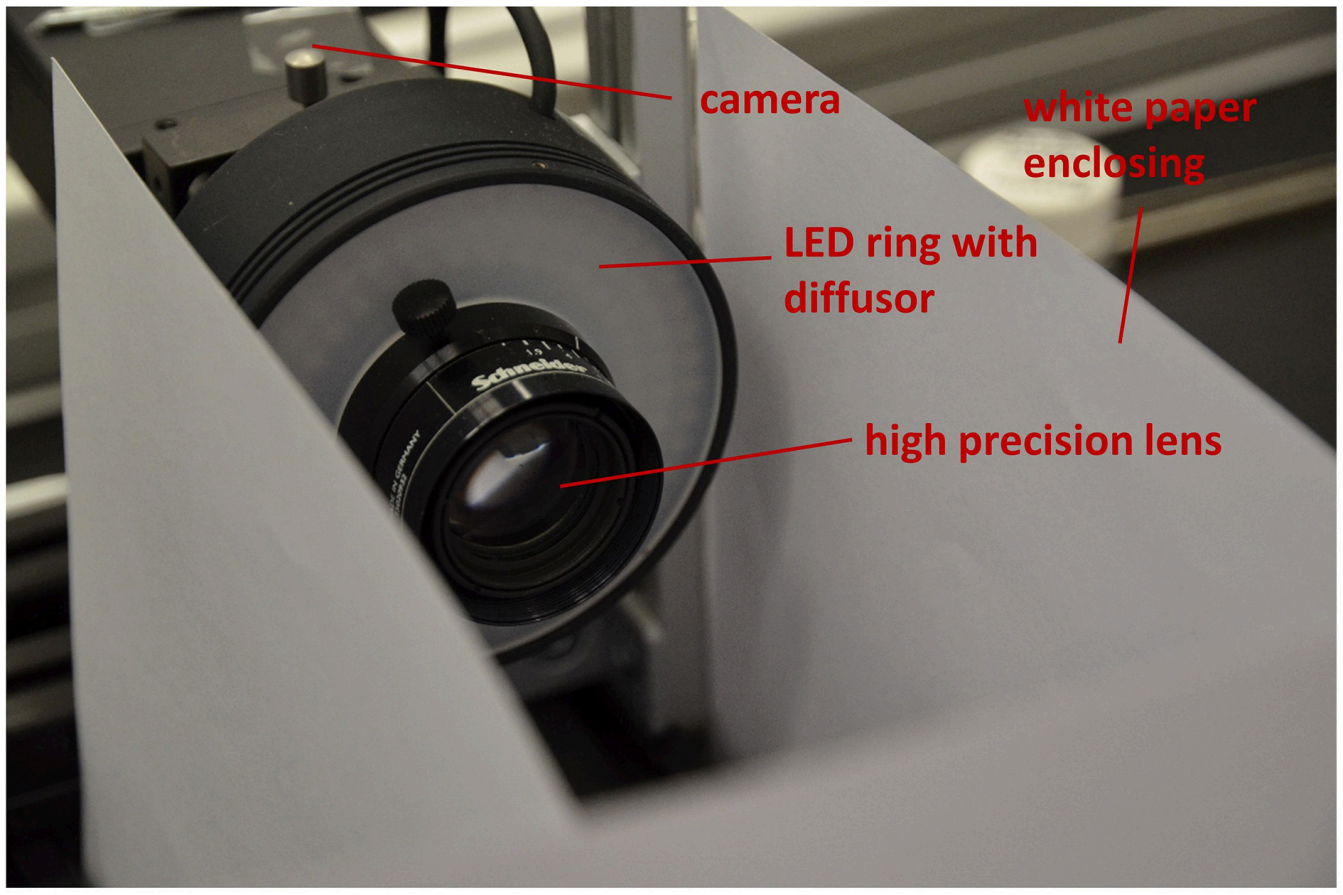
**Camera setup for 3D imaging**.

Depending on seed size for 3D reconstruction we use two different setups for image acquisition. Both setups consist of an industrial-grade c-mount camera (PointGrey Grasshopper, GRAS-50S5M-C, Mono, 5.0 MP, Sony ICX625 CCD, 2/3", square pixels of size μ = 3.45 μm, global shutter, 2448 × 2048, 15 FPS), 35 mm high precision lens (Schneider KMP APO-XENOPLAN 35/1,9) and a white LED ring with diffusor (CCS LDR2-70-SW2) shown in Figure 4. For small seeds (e.g., *Arabidopsis*, tobacco, rapeseed) a 36 mm spacer is mounted between camera and lens. For larger seeds (e.g., barley, maize) only a 15 mm spacer is needed. Spacer reduce the minimum working distance of the lens (*d* = 69.9 mm for the 36 mm spacer, 128.0 mm for the other) and thus are responsible for suitable magnification. This allows to measure seeds in a range between ≈0.2 and 12 mm. White paper is used as background.

For image acquisition seeds are picked by a cone-shaped vacuum nozzle and held in front of the camera at optimal working distance using a robotic system to be described elsewhere. The robot rotates the seed in configurable angles and triggers the camera. We use 10° steps and take 36 images, if not stated differently. Image acquisition times are mainly limited by the robot's rotation typically ≳ 2.7 − 5*s* per 360°, depending on the motion type. We perform 36 stop-and-go steps resulting in an overall acquisition time of ≈6 − 7 s.

### 3.2. Camera calibration

We use the OpenCV implementation (Bradski and Kaehler, 2008) of Bouguet's calibration method (Bouguet, 1999) and an asymmetric 4 × 11 dot-pattern target with a total size of 5.8 × 4.3 mm. It was printed using a professional, high resolution film recorder, as usual office printers even on good paper do not achieve a printing precision suitable for camera calibration at such small spatial scales.

Using this toolbox, estimation of the focal length *f* is not precise enough for our purposes. We therefore use a ball-bearing ball (steel, precision according to DIN5401 G20) with *r*_0_ = 1.50 mm ±0.25 μm radius as calibration object, in order to estimate the working distance *d* (or equivalently focal length *f* from working distance *d*) of our system precisely. From a mask image of the ball acquired with our system, we estimate its area *A* in pixel. This allows to estimate its radius *r* in the image by $r=\mu \sqrt{A∕\pi }$, where μ is the pixel size. From basic geometric reasoning working distance *d* can be derived as $d=\sqrt{f^{2}+r^{2}}r_{0}∕r$.

### 3.3. Software implementation

The software framework is implemented in C++ on a Windows 7 operating system with Visual Studio 2013. The application programming interface Open Graphics Library (*OpenGL* OpenGL.org, 2015) was used for the GPU implementation.

As supplemental material (Roussel et al., 2016) we provide both, a suite of *Matlab* (Mathworks, 2015) routines as well as a Python implementation suitable for volume carving of not too small seeds using an affordable imaging setup. Such setups may e.g., use a turntable and a consumer SLR camera. In our example (see Figure 2) we use normalized cross-correlation for image registration, as the bottom of the tool is always visible as a suitable registration target.

## 4. Experiments

### 4.1. Resolution and runtime

The complexity of the volume carving algorithm is proportional to the number *N*_*V*_ of voxels and number *N* of images acquired. For our equidistantly spaced cubic *R* × *R* × *R* grids the voxel number is $N_{V}=R^{3}$ and thus complexity is *O*(*R*^3^*N*). In addition time for loading (or acquiring) the images (with *N*_*P*_ pixels) and, for the GPU implementation, transferring the data to and from the graphics card is needed. Complexity of this data transfer and preprocessing of the images is *O*(*N*_*P*_*N*), or $O\left(N_{P}N\right)+O\left(R^{3}\right)$ for the GPU implementation.

**Figure 5:**
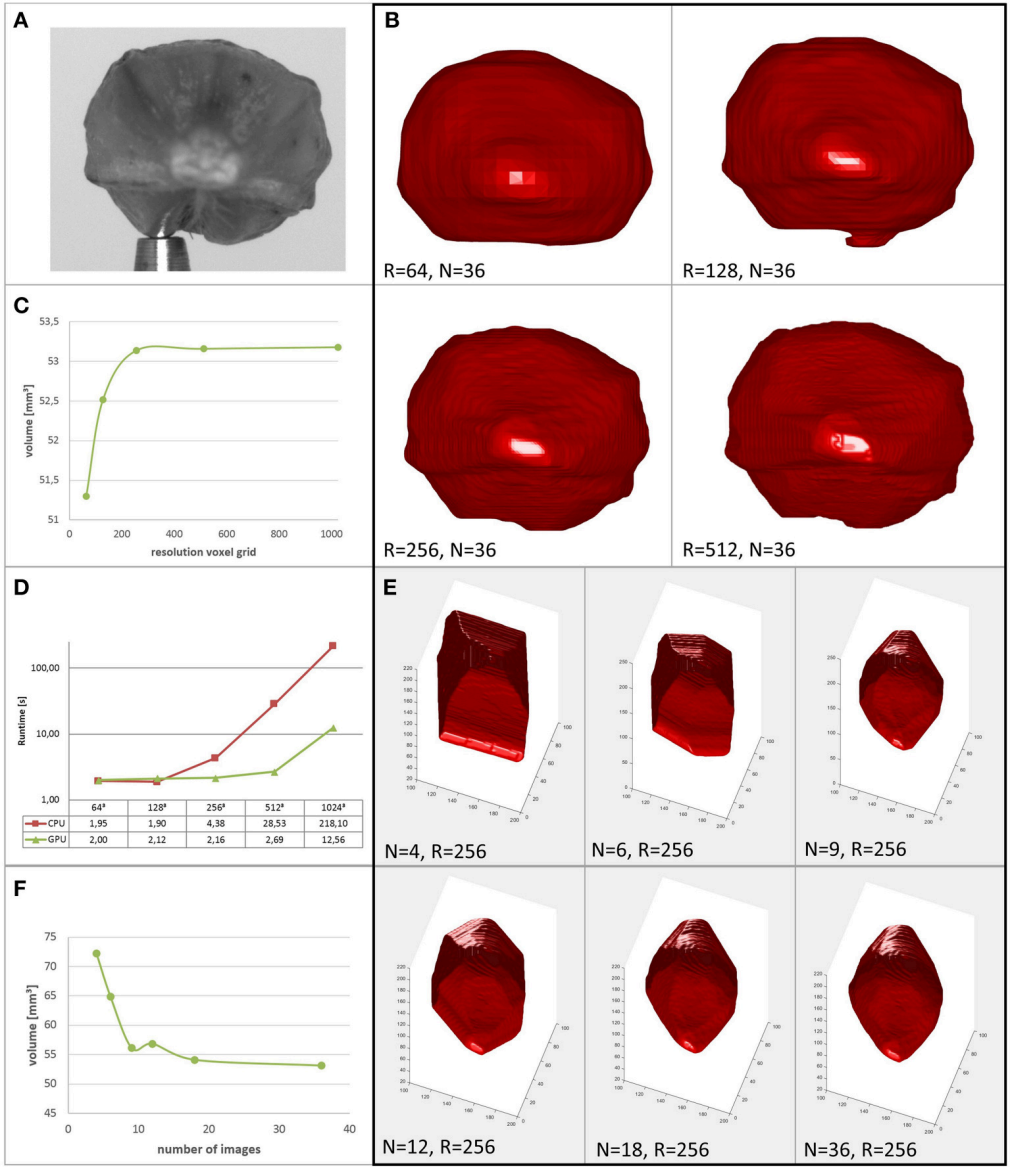
**Performance of the proposed method**. **(A)** Original image of a barley seed. **(B)** Reconstructions of the seed at different grid resolutions. **(C)** Reconstructed volume vs. resolution of the voxel grid. **(D)** Runtimes in seconds of serial CPU and parallel GPU implementations (reproduced from Brenscheidt, 2014). **(E)** Reconstructions of the seed using different numbers of images. **(F)** Reconstructed volume vs. number of images used.

Runtimes shown in Figure 5D have been measured on a PC with Intel Core i5-3470 CPU, 8GB DDR3 RAM and an NVIDIA GeForce GTX 580 GPU with 4047MB GDDR5 RAM (*cmp*. Brenscheidt, 2014 for further details). We observe that for low resolutions *R* of the voxel grid, runtime contributions by the *O*(*N*_*P*_*N*) components dominate, as no dependence on *R* is visible. For increasing *R*, these parts become negligible. While for the CPU implementation a significant increase of the runtime vs. the 2 s runtime for smallest voxel resolutions can be noticed at *R* = 256 (4s), the parallel GPU implementation stays at comparable runtimes even at *R* = 512.

When interested in a seed's volume as a trait used for high throughput phenotyping, rather than in subtle surface details, voxel resolution can be selected comparably low. In Figures 5A–C we show a barley seed and its reconstructions together with its derived volume for different grid resolutions *R*. We observe that above *R* = 256 the estimated volume is approximately constant. Thus, for this phenotyping task, runtime is limited mainly by file-IO, transfer and preprocessing. Sophisticated speed-up mechanisms for the carving provide rather low benefits in this application, as their main potential lies in higher achievable volume resolutions.

Speedup using fewer images may be paid by lower accuracy (see Section 4.2). We show reconstructions of the same barley seed in Figure 5E and the corresponding volumes in Figure 5F. Images are selected equidistantly. We observe that reducing image number rapidly reduces reconstruction quality. Interesting to note is that the reconstruction using *N* = 9 images is more accurate than with *N* = 12 images. This is due to the fact that for *N* = 12 the selected angle between images is α = 30°, thus 180° is a multiple of α (the same is true for *N* ∈ {4, 6, 12, 18, 36}, *cmp*. also **Figure 8** and Section 4.2). However, as the opening angle of our lens is small, complementary information content in masks coming from cameras looking in opposite direction is low. We conclude that for shorter runtimes with comparable or even higher reconstruction accuracy investigating alternative viewing directions is promising. We do this in the next section for the restricted possibilities of our robotic, turntable-like, single camera acquisition system.

### 4.2. Accuracy vs. number of images

**Figure 6:**
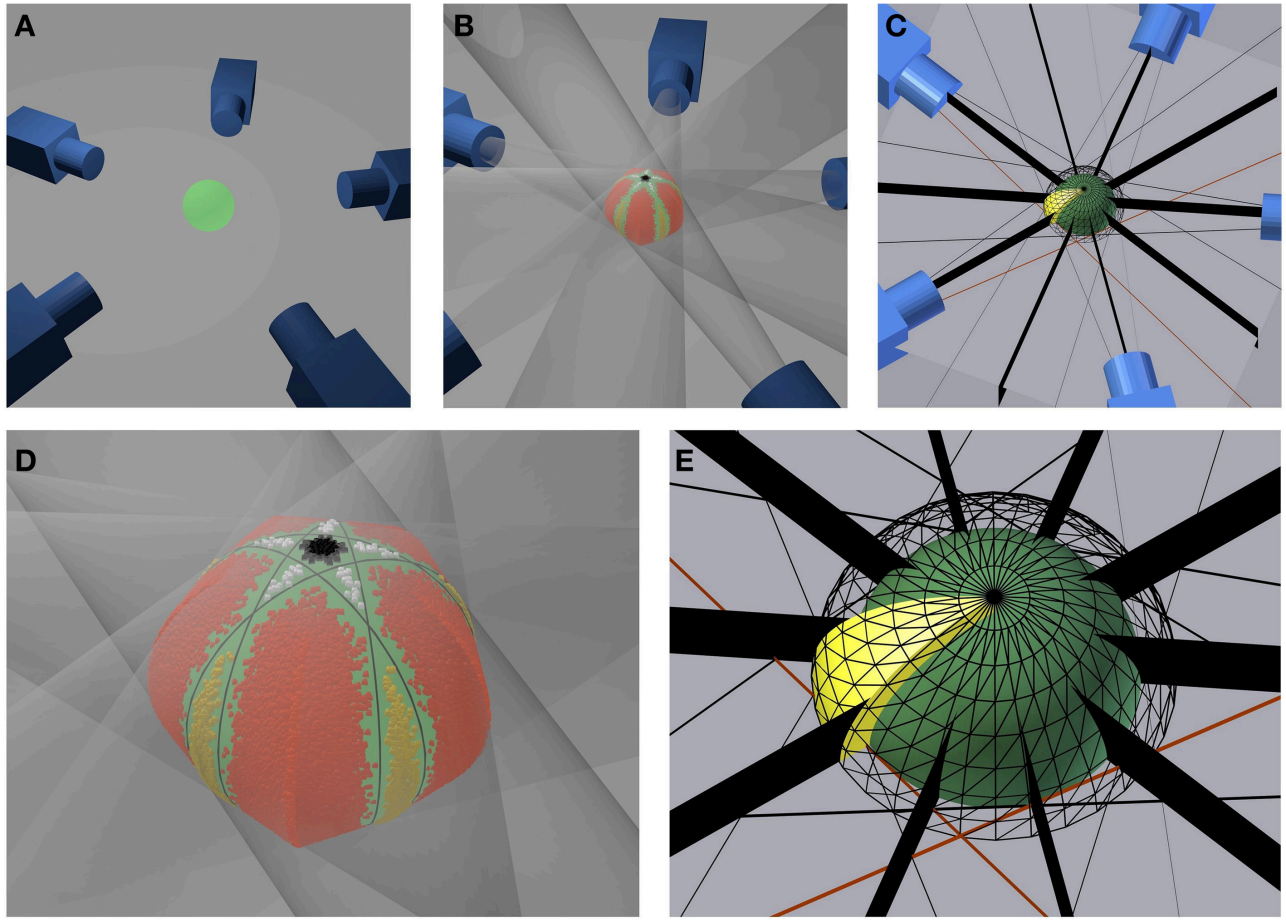
**Setup for the numerical accuracy analysis**. **(A)** Exemplary geometrical setup for five cameras. **(B)** Same configuration with transparent projection cones touching the green sphere and colored inlier sampling points. **(D)** Close up of the sphere (green) and a visualization of the sampled inlier points. Red, yellow, black, and white points indicate the four different types if surplus volumes not taken away by carving. Black lines indicate where cones touch the sphere **(C)** Symmetry planes (black) for each camera and section of a spherical shell indicating the geometry of the sampling region (yellow). **(E)** Close up of the sampling region (yellow). The inner sphere (green) represents the inner border of the volume, the meshed sphere the outer border. Black stripes indicate symmetry planes.

We numerically investigate the influence of the number *N* of equidistantly acquired images on the accuracy of volume estimation in an ideal turntable setting. To do so, we calculate the volume *V*_num_ of a sphere with radius *r*_0_ derived as cut of tangent cones, cmp. Figures 6A,B,D: Each ideal camera is represented by its camera center *C*_*i*_, known from the selected working distance and rotation angle. A sphere projects to a circle on the sensor plane. A volume carving step for each image of this sphere thus corresponds to testing for each point of the working volume, if it lies inside or outside a cone spanned by *C*_*i*_ and the outline of the projected sphere (transparent cones in Figure 6B). The cone is independent of the focal length *f* of the ideal camera, but depends on the working distance *d*, i.e., the distance between each camera center and the center of the sphere. We select *r*_0_ = 1.5 mm, as we use a highly accurate, spherical bearing ball of this size as ground truth object in real experiments (see Section 4.4).

As we did not find a closed form solution for the volume of an object derived by a cut of *N* cones for arbitrary *N*, we numerically integrate the volume by a Monte-Carlo method:

1. We randomly select *K* points in a region with known volume *V*_reg_ including the complete test volume.
2. For each point we test, if it lies in *all* cones spanned by the cameras. If yes, the point lies in the volume, if no, not. The number of all inliers is *K*_in_.
3. The sought-for volume *V*_num_ is then *V*_num_ ≈ *V*_reg_^*^ *K*_in_∕*K*.

The smaller *V*_reg_ can be selected, the more accurate *V*_num_ can be approximated with a fixed number *K* of sampling points. We observe that the selected turntable camera configuration (Figures 6A,C) is symmetric with respect to

- the plane spanned by the cone centers i.e., the equator of the sphere,
- each plane spanned by the rotation axis and a camera center,
- each plane spanned by the rotation axis and cutting the rotation angle between two adjacent camera centers in half, i.e., the vertical plane between two cameras.

Furthermore, we observe that

- the inner of the sphere lies completely in the carved volume, i.e., we carve the sphere from the outside; and finally that
- the sought-for volume lies in a concentric sphere with a somewhat larger radius than the carved sphere.

This allows to restrict numerical calculations to a region with known volume *V*_reg_ being box interval in spherical coordinates (*cmp*. Figure 6E, yellow region), in order to benefit optimally from symmetries in the problem. We restrict the altitude angle to θ ∈ [0, π∕2], azimuth to ϕ ∈ [0, π∕(2*N*)], and the radius to *r* ∈ [*r*_0_, *r*_1_]. Radius *r*_1_ is calculated by (1) intersecting all cones with the plane spanned by the camera centers, yielding a pair of lines for each cone, (2) selecting the right lines of two adjacent cones (*cmp*. example red lines in Figures 6C,E) (3) calculating the intersection between these lines, and (4) selecting *r*_1_ as radius of the point given in polar coordinates.

This geometry is depicted in Figures 6C,E. The selection correspond to one section of a spherical shell, cut in half by the plane spanned by the cone centers (i.e., at the equator of the sphere) and cut in 2*N* sections by the half-planes starting at the axis of rotation symmetry (i.e., the axis through the poles of the sphere) and each including one cone center; as well as their angle bisector planes.

Randomly sampling points in spherical coordinates produces higher point densities toward the origin in Euclidean coordinates and toward the north-south-axis of the sphere. We correct for these density differences combining two approaches. To understand this we need to calculate the Jacobian of the spherical coordinate transformation from Euclidean coordinates. Using the convention

(4) $$\begin{array}{lll} x & =r\text{ sin }\theta \text{ cos }\phi \; & \; \end{array}$$

(5) $$\begin{array}{lll} y & =r\text{ sin }\theta \text{ sin }\phi \; & \; \end{array}$$

(6) $$\begin{array}{lll} z & =r\text{ cos }\theta \; & \; \end{array}$$

we get the Jacobian *J*

(7) $$\begin{array}{l} J=\det\frac{\partial (x,y,z)}{\partial (r,\theta ,\phi )}\\ \;=\det\left(\begin{array}{ccc} \sin\theta \cos\phi & r\cos\theta \cos\phi & -r\sin\theta \sin\phi \\ \sin\theta \sin\phi & r\cos\theta \sin\phi & r\sin\theta \cos\phi \\ \cos\theta & -r\sin\theta & 0 \end{array}\right)=r^{2}\sin\theta . \end{array}$$

As long as *r*_0_ and *r*_1_ do not differ too much, as in our case, it is sufficient to consider the radial part of *J* by weighing sampling points *p* by their radius value *r*_*p*_

(8) $$\begin{array}{l} V_{\text{num}}\approx V_{\text{reg}}*{\tilde{K}}_{\text{in}}∕\tilde{K} \end{array}$$

where ${\tilde{K}}_{\text{in}}=\underset{p\in P_{\text{in}}}{\sum }r_{p}^{2}$ and $\tilde{K}=\underset{p\in P}{\sum }r_{p}^{2}$. *P* is the set of all sampling points and *P*_in_⊂*P* the set of all inliers.

Density variations due to altitude θ are compensated by transforming the sampling probability density from the uniform distribution *p*(χ) (with χ ∈ [0, 1]) a random number generator delivers to a sin(θ)-shaped distribution *p*_θ_(θ). This is achieved by the transform θ = *g*(χ), where *g*(χ) = arccos(1 − χ). This can be easily verified using to the transform law for densities $p_{\theta }\left(\theta \right)=|\frac{d}{d\theta }g^{-1}\left(\theta \right)|p\left(g^{-1}\left(\theta \right)\right)$, where *g*^−1^ denotes the inverse function of *g*.

**Figure 7:**
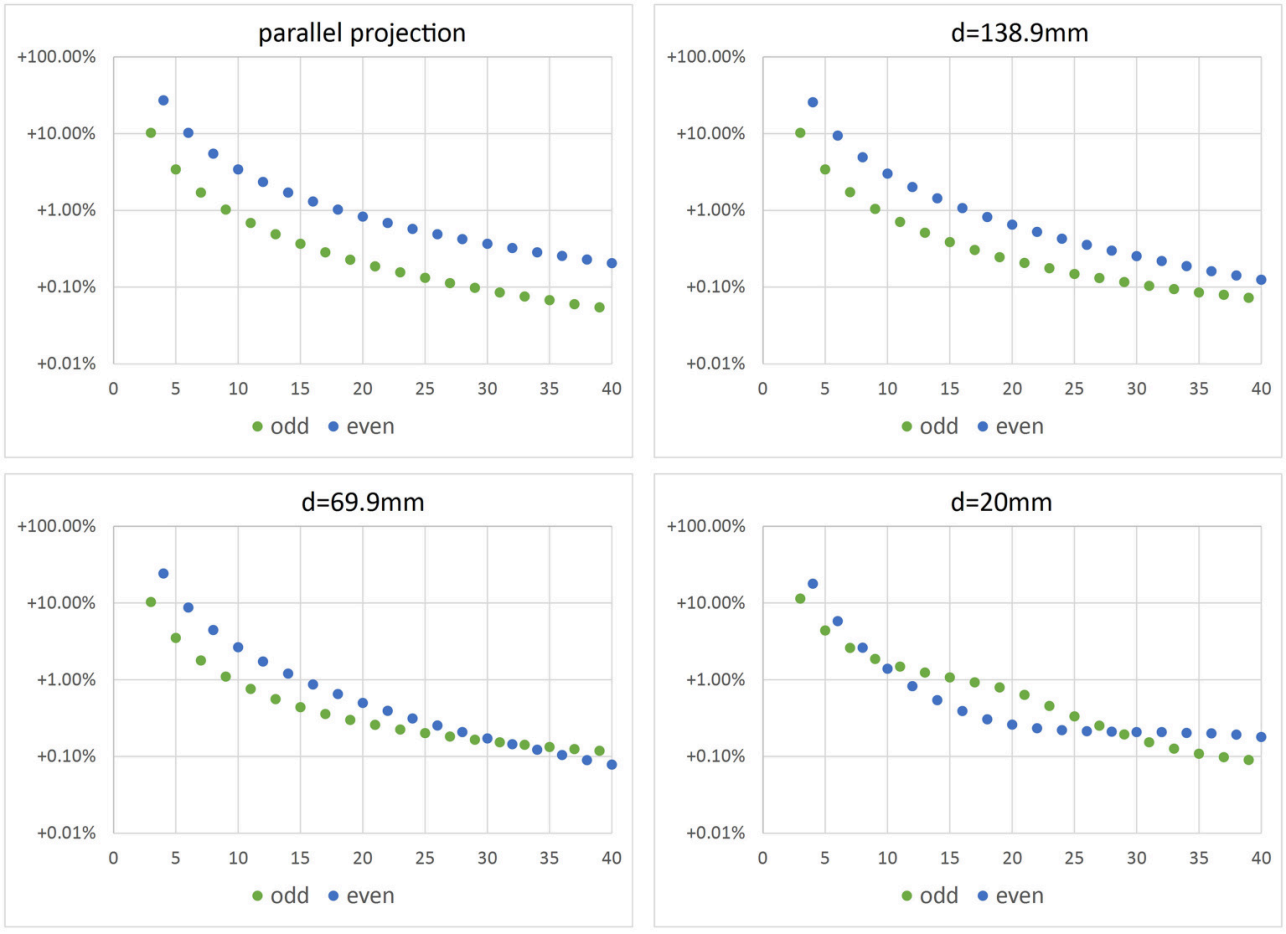
**Error of volume vs. number ***N*** of camera positions**. Top left: error when using parallel projection (telecentric lens). The other plots show errors for different working distances *d* and central projection. In real experiments we use working distance *d* = 69.9 mm.

In Figure 7 relative error *E* = (*V*_num_ − *V*_0_)∕*V*_0_ with $V_{0}=4∕3\pi r_{0}^{3}$ is depicted vs. the number *N* of images used for reconstruction of *V*_num_. We observe that for parallel projection, using an even number of cameras (or images) yields the same result as using half the number of images. This makes sense, as for an even number of cameras, pairs of cameras are in an 180°-configuration, looking at the same object contour from different sides. This does not add additional information to the reconstruction. However, when considering central projection also images from an 180°-configuration add additional information, as cameras do not look at the same contour.

**Figure 8:**
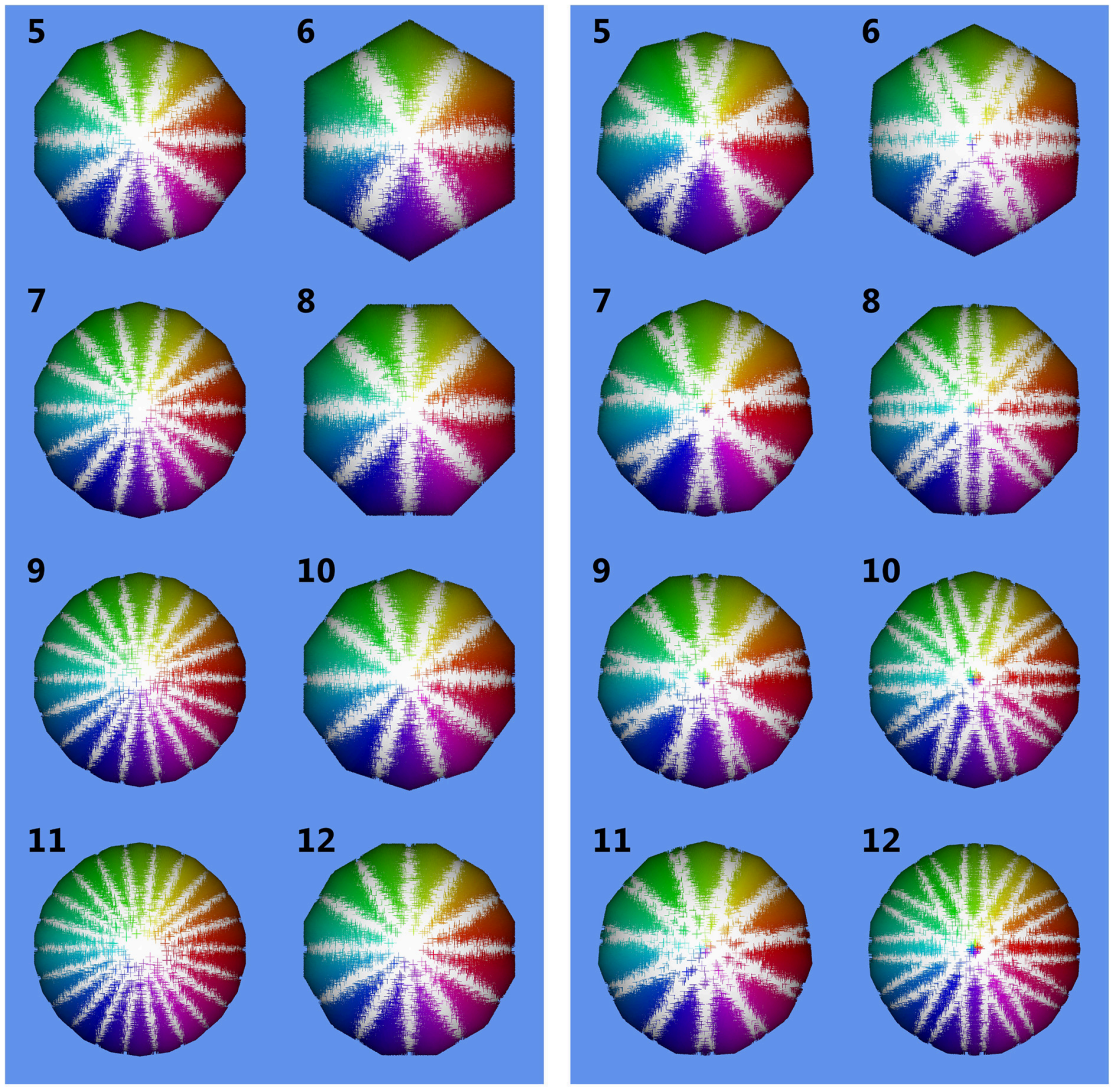
**Reconstructed volumes for different numbers ***N*** of camera positions**. Numbers in the upper left corner of each subimage are *N*. Inlier sampling points are color-coded, where hue indicates ϕ and brightness θ. The underlying ground truth sphere is white. **Left:** reconstruction using parallel projection (telecentric lens). **Right:** reconstruction using central projection with working distance *d* = 20*mm* and sphere radius *r*_0_ = 1.5 mm.

Figure 8 shows volumes reconstructed using different camera configurations. We observe that using *N* = 5 or *N* = 10 camera positions yields identical results for parallel projection in Figure 8, left. However, for central projection *N* = 10 camera positions yields a much better reconstruction than using *N* = 5 images. This is in full agreement with the plot given in Figure 7, bottom right, where we also observe, that using odd *N* is not always better than using even *N* with one additional camera position. It depends on object size in relation to working distance, which camera position configuration yields better results in theory. We observe, that in the cases tested here, a minimum of *N* = 11 or *N* = 12 images is needed to stay below 1% relative error. A limit of less than 0.1% error is reached using *N* = 33, *N* = 36, or *N* = 37 images. In practice, however, given a sufficiently high *N*, other error sources may dominate (see next Sections).

### 4.3. Accuracy loss due to position inaccuracies

We performed experiments using synthetic images in order to test the different error sources in our method. The images showed a perfect sphere as projection of a synthetic ball of same size as our bearing-ball. Geometrical setup was as for the experiments in Section 4.2, with working distance *d* = 69.9 mm. We reconstructed the ball using volume carving, a voxel grid resolution of 256^3^ and (12 μm)^3^ voxel size. Results were very similar to the ones shown in Figure 7 for the same *d*, e.g., relative error at 36 images was approximately 0.14% instead of 0.1%. We do not show this plot. We conclude, that volume carving on a fine enough grid comes close to the theoretical performance of the simulation from Section 4.2, in agreement with our observations in Figure 5C.

**Figure 9:**
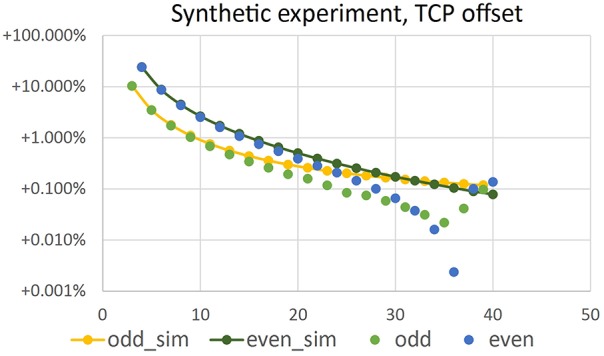
**Absolute value of relative error of volume vs. number ***N*** of camera positions in a synthetic volume carving experiment, where an offset of 7 pixel has been introduced in ${\vec{x}}_{\text{TCP}}$ of the first image**. Configuration is identical to the simulation results shown in Figure 7. Yellow and dark green points have been repeated from there.

Estimating TCP locations in the images is critical for reconstruction accuracy. To test the influence of a TCP wrongly located in an image, we add a relatively large offset of 7 pixel in *y*-direction to ${\vec{x}}_{\text{TCP}}$ in the first image and leave the TCP locations in all other images untouched. The carving result is shown in Figure 9. The volume loss due to the misaligned TCP is in the order of 0.001 to 0.002 mm^3^, and thus reduces the positive volume error due to non-carved regions (as described in Section 4.2). For image configurations with relatively low positive initial error, i.e., here *N* > 36, the error due to the TCP offset dominates, such that overall relative error is negative. Please note that Figure 9 shows absolute values of the relative errors due to the log-scale.

### 4.4. Seed types and overall accuracy

**Figure 10:**
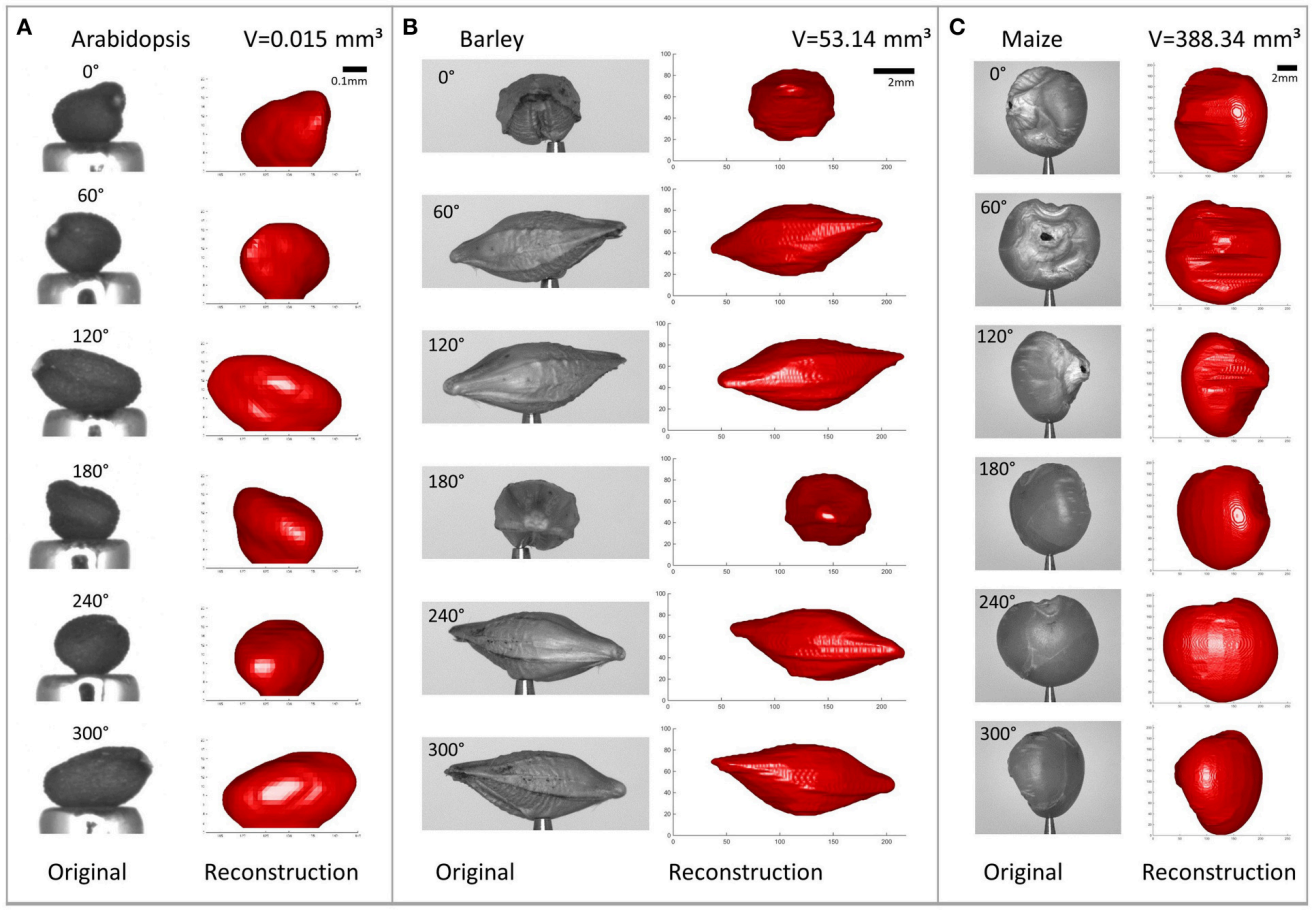
**Reconstructed seeds shown from different angles, side-by-side with the original images (***R*** = 256, ***N*** = 36)**. **(A)** *Arabidopsis*. **(B)** Barley. **(C)** Maize. Please note the different scalings.

Using the proposed method we reconstructed different seed types, namely *Arabidopsis* (length, i.e., longest dimension ≲ 0.5 mm), rapeseed (≈ 2 mm), barley (≈ 8 mm), and maize (≈ 11 mm). See Figure 10 to get an impression of the usually achieved reconstruction accuracy.

Absolute accuracy is validated experimentally using two different test objects. The first one is the ball-bearing ball we used for working distance calibration with 3.00 mm ±0.5 μm diameter, i.e., 0.02% diameter tolerance, and thus a precisely known volume of 14.137 mm^3^ ±0.007 mm^3^, i.e., 0.05% volume tolerance. Clearly, as we used this object for calibration, scaling of the mask images exactly fit to the respective projection matrices **P**_*i*_. However, the overall performance of the system for volume reconstruction can still be evaluated using this object, as the volume derived has *not* been used for calibration and still accumulates all errors and imperfections the method has. The second object is an ink cartridge ball with 2.45 mm ±0.02 mm diameter, i.e., 0.4% diameter measurement error, measured with a digital sliding caliper, and thus a volume of 7.70 mm^3^± 0.19 mm^3^, i.e., 2.5% volume error.

**Figure 11:**
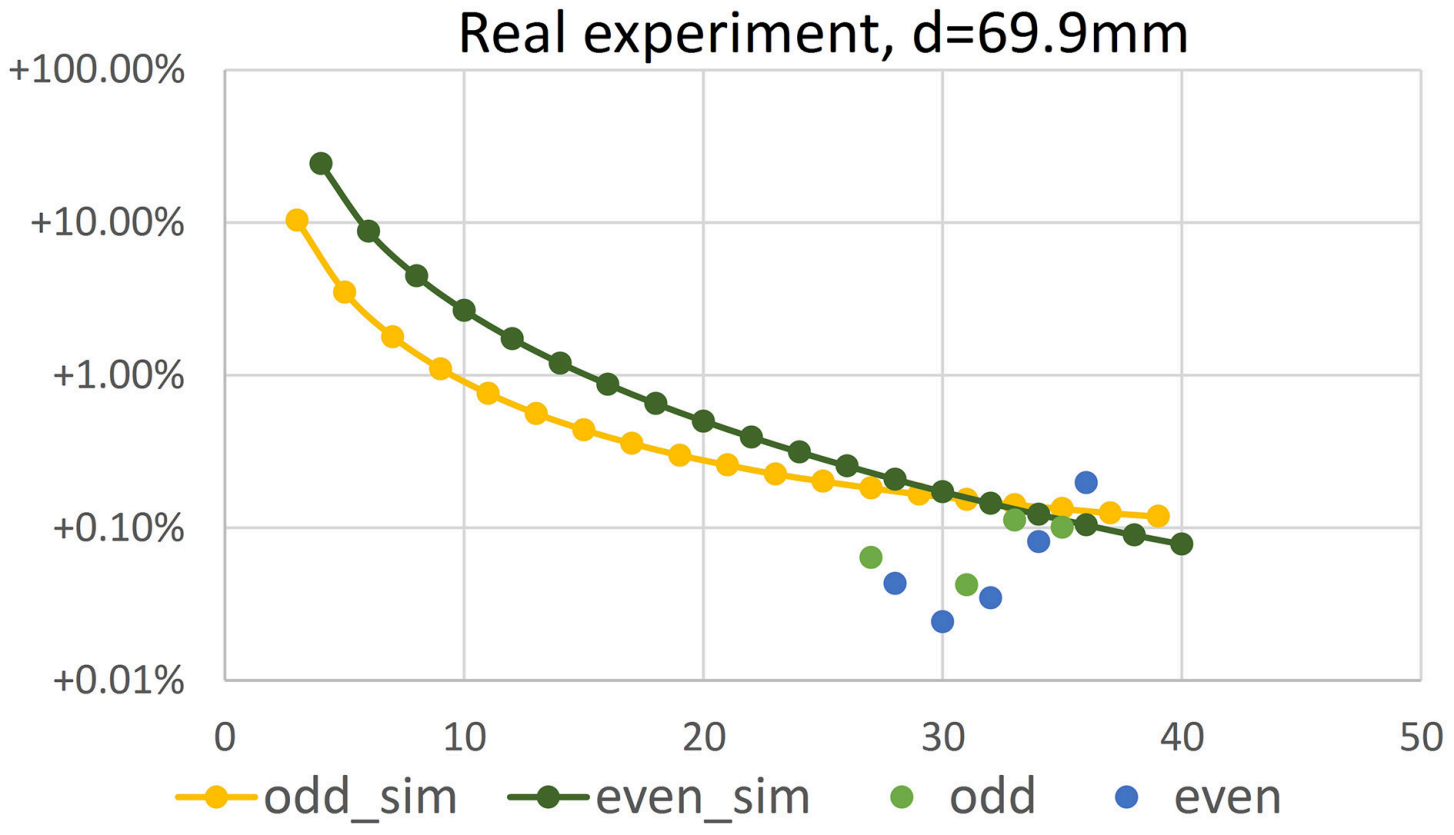
**Error of volume vs. number ***N*** of camera positions in a real experiment using a bearing ball**. Configuration is identical to the simulation results shown in Figure 7, repeated in the plot for reference.

In Figure 11 volume error vs. number *N* ∈ {27, …, 36} of imaging positions is shown for the ball-bearing ball experiment. Comparing results to Figure 7, lower left (values repeated in Figure 11 for easy reference), we observe that negative reconstruction errors due to positioning inaccuracies are in the same range as the positive theoretical errors and increase with increasing number of images. Please note that the plot shows absolute values of relative errors, as negative errors can not be displayed in log-scale.

Reconstructing the ball using 36 images as used for seed reconstruction, a voxel grid resolution of 256^3^ and (12 μm)^3^ voxel size, i.e., a volume quite tightly surrounding the object, yields a volume of 14.11 mm^3^ and thus a mean diameter of 2.998 mm for the bearing ball. This is a relative error of −0.19% wrt. the specified volume, and of −0.06% wrt. the diameter, when calculating the diameter from the measured volume, assuming a perfect sphere. For the ink cartridge ball we measure 7.83 mm^3^ corresponding to a mean diameter of 2.46 mm, being well within the measuring error of our caliper measurement.

We conclude that the overall accuracy of our method, including camera calibration error, mechanical imperfections, TCP finding error, imprecision due to the simple carving approach etc. is high enough to compete with or even beat a precise slide caliper for length measurements. Absolute values of measurement errors of volume and lengths are in the range of few per mill.

## 5. Conclusion and outlook

Simple volume carving combined with a method for extrinsic camera pose estimation from images is sufficiently accurate for size measurements of even tiny seeds. To optimize our system for runtime and accuracy, we investigated its performance using different parameter settings. Surprisingly, the main performance gain potential does currently not lie in using more sophisticated reconstruction methods allowing for higher achievable voxel resolutions *R*, e.g., achievable by the highly accurate method presented by Klodt and Cremers (2015) and necessary for reconstruction of more complex surfaces. Our findings allow reducing preprocessing and transfer times by selecting a suitable image number *N* and comparably low voxel resolution of 256^3^.

The optimal number *N* of acquisition positions used in a turntable setting depends on the selected projection geometry. In our case using *N* = 36 images yields a theoretical overestimation of the reconstructed volume of a sphere by +0.1% relative error.

The method's achievable accuracy has been tested experimentally using a highly accurate spherical object. Systematic errors are much lower than we expected, between +0.06% for *N* = 27 and −0.19% for *N* = 36. This means that volume losses due to inaccurate positioning of the object are truly negligible for our purposes. Clearly, as seeds are not well represented by a ball, such accuracy studies give insight in the accuracy *potential* of the method—if it fails on a ball, it will also fail on more complex shapes.

Seed-shape-specific errors are not well captured by a ball and may vary from seed type to seed type. Alternative simple volume measurement methods for ground truthing, e.g., Archimedes' principle, are not accurate enough for such small objects, but high-resolution CT may be an option.

Many factors influence the accuracy, e.g., segmentation errors, small dust particles or camera pose errors. Most critical are inaccuracies of ${\vec{x}}_{\text{TCP}}$ in the image, leading to parts of a seed being erroneously carved away. To detect such errors, suitable error estimation methods can be implemented to complement the method proposed here, e.g., summing back-projection error. When positioning accuracy is an issue, more elaborated, iterative, optimization-based but also more costly to calculate methods may be applied. However, as we have seen in our experiments, achieved accuracies are well high enough for seed phenotyping even without such corrections.

Overall we conclude that the presented method yields highly accurate seed reconstructions being accurate enough when interested in seed volume.

## Author contributions

SJ and HS designed the robotic system used for seed handling and image acquisition. JR and AF implemented robot and acquisition hard and software. JR performed all lab experiments. FG performed all numerical experiments and implemented the supplemental Python code. HS implemented the baseline method, the supplemental Matlab code, and drafted the manuscript. All authors contributed to text and figures of the manuscript and approved the final manuscript.

### Conflict of interest statement

The authors declare that the research was conducted in the absence of any commercial or financial relationships that could be construed as a potential conflict of interest.

## References

[B1] Aksoy E. E. Abramov A. Wörgötter F. Scharr H. Fischbach A. Dellen B. (2015). Modeling leaf growth of rosette plants using infrared stereo image sequences. Comput. Electron. Agric. 110, 78–90. 10.1016/j.compag.2014.10.020

[B2] Alenya G. Dellen B. Torras C. (2011). 3D modelling of leaves from color and ToF data for robotized plant measuring, in IEEE International Conference on Robotics and Automation (Shanghai), 3408–3414. 10.1109/icra.2011.5980092

[B3] Arvidsson S. Pérez-Rodríguez P. Mueller-Roeber B. (2011). A growth phenotyping pipeline for *Arabidopsis thaliana* integrating image analysis and rosette area modeling for robust quantification of genotype effects. New Phytol. 191, 895–907. 10.1111/j.1469-8137.2011.03756.x 21569033

[B4] Augustin M. Haxhimusa Y. Busch W. Kropatsch W. G. (2015). Image-based phenotyping of the mature arabidopsis shoot system, in Computer Vision - ECCV 2014 Workshops, Vol. 8928 of *Lecture Notes in Computer Science* (Zurich: Springer International Publishing), 231–246.

[B5] Bouguet J.-Y. (1999). Visual Methods for Three-dimensional Modeling. Ph.D thesis, California Institute of Technology, Pasadena, CA, USA.

[B6] Bradski G. Kaehler A. (2008). Learning OpenCV. Sebastopol, CA: O'Reilly Media.

[B7] Brenscheidt M. (2014). Rekonstruktion der visuellen Hülle von Pflanzensamen mithilfe der OpenGL. Bachelor's thesis, Fachhochschule Aachen Campus Jülich, Germany.

[B8] Brewer M. T. Lang L. Fujimura K. Dujmovic N. Gray S. van der Knaap E. (2006). Development of a controlled vocabulary and software application to analyze fruit shape variation in tomato and other plant species. Plant Physiol. 141, 15–25. 10.1104/pp.106.077867 16684933 PMC1459328

[B9] Bylesjö M. Segura V. Soolanayakanahally R. Y. Rae A. M. Trygg J. Gustafsson P.. (2008). Lamina: a tool for rapid quantification of leaf size and shape parameters. BMC Plant Biol. 8:82. 10.1186/1471-2229-8-82 18647399 PMC2500018

[B10] Clark R. T. MacCurdy R. B. Jung J. K. Shaff J. E. McCouch S. R. Aneshansley D. J.. (2011). Three-dimensional root phenotyping with a novel imaging and software platform. Plant Physiol. 2, 455–465. 10.1104/pp.110.169102 21454799 PMC3177249

[B11] De Vylder J. Ochoa D. Philips W. Chaerle L. Van Der Straeten D. (2011). Leaf segmentation and tracking using probabilistic parametric active contours, in International Conference on Computer Vision/Computer Graphics Collaboration Techniques (Rocquencourt), 75–85.

[B12] Dellen B. Scharr H. Torras C. (2015). Growth signatures of rosette plants from time-lapse video. IEEE/ACM Trans. Comput. Biol. Bioinf. 12, 1470–1478. 10.1109/tcbb.2015.2404810 26684463

[B13] Denso Robotics Europe (2015). Denso Main Brochure. EN_Global_EU_042015_V1, Accessed June 2015.

[B14] Dice L. (1945). Measures of the amount of ecologic association between species. Ecology 26, 297–302. 10.2307/1932409

[B15] Everingham M. Van Gool L. Williams C. K. I. Winn J. Zisserman A. (2010). The Pascal Visual Object Classes (VOC) challenge. Int. J. Comput. Vis. 88, 303–338. 10.1007/s11263-009-0275-4

[B16] Fahlgren N. Gehan M. A. Baxter I. (2015). Lights, camera, action: high-throughput plant phenotyping is ready for a close-up. Curr. Opin. Plant Biol. 24, 93–99. 10.1016/j.pbi.2015.02.006 25733069

[B17] Fang S. Yan X. Liao H. (2009). 3d reconstruction and dynamic modeling of root architecture *in situ* and its application to crop phosphorus research. Plant J. 60, 1096–1108. 10.1111/j.1365-313X.2009.04009.x 19709387

[B18] Golbach F. Kootstra G. Damjanovic S. Otten G. Zedde R. (2015). Validation of plant part measurements using a 3d reconstruction method suitable for high-throughput seedling phenotyping. Mach. Vis. Appl. [Epub ahead of print]. 10.1007/s00138-015-0727-5.

[B19] Granier C. Aguirrezabal L. Chenu K. Cookson S. J. Dauzat M. Hamard P.. (2006). PHENOPSIS, an automated platform for reproducible phenotyping of plant responses to soil water deficit in *Arabidopsis thaliana* permitted the identification of an accession with low sensitivity to soil water deficit. New Phytol. 169, 623–635. 10.1111/j.1469-8137.2005.01609.x 16411964

[B20] Hartley R. I. Zisserman A. (2004). Multiple View Geometry in Computer Vision, 2nd Edn. Canberra: Cambridge University Press.

[B21] Hartmann A. Czauderna T. Hoffmann R. Stein N. Schreiber F. (2011). HTPheno: an image analysis pipeline for high-throughput plant phenotyping. BMC Bioinform. 12:148. 10.1186/1471-2105-12-148 21569390 PMC3113939

[B22] Herridge R. P. Day R. C. Baldwin S. Macknight R. C. (2011). Rapid analysis of seed size in *Arabidopsis* for mutant and QTL discovery. Plant Methods 7, 3. 10.1186/1746-4811-7-3 21303553 PMC3046896

[B23] Iwata H. Ebana K. Uga Y. Hayashi T. Jannink J.-L. (2010). Genome-wide association study of grain shape variation among oryza sativa l. germplasms based on elliptic fourier analysis. Mol. Breeding 25, 203–215. 10.1007/s11032-009-9319-2

[B24] Iwata H. Ukai Y. (2002). SHAPE: a computer program package for quantitative evaluation of biological shapes based on elliptic fourier descriptors. J. Hered. 93, 384–385. 10.1093/jhered/93.5.384 12547931

[B25] Jansen M. Gilmer F. Biskup B. Nagel K. Rascher U. Fischbach A. (2009). Simultaneous phenotyping of leaf growth and chlorophyll fluorescence via GROWSCREEN FLUORO allows detection of stress tolerance in *Arabidopsis thaliana* and other rosette plants. Funct. Pant Biol. 36, 902–914. 10.1071/FP09095 32688701

[B26] Klodt M. Cremers D. (2015). High-resolution plant shape measurements from multi-view stereo reconstruction, in Computer Vision - ECCV 2014 Workshops, Vol. 8928 of Lecture Notes in Computer Science, eds Agapito L. Bronstein M. M. Rother C. (Zurich: Springer International Publishing), 174–184.

[B27] Koenderink N. J. J. P. Wigham M. Golbach F. Otten G. Gerlich R. van de Zedde H. J. (2009). MARVIN: high speed 3d imaging for seedling classification, in Seventh European Conference on Precision Agriculture 2009 (Wageningen), 279–286.

[B28] Laurentini A. (1994). The visual hull concept for silhouette-based image understanding. Pattern Anal. Mach. Intell. IEEE Trans. 16, 150–162. 10.1109/34.273735

[B29] Lobet G. Draye X. Périlleux C. (2013). An online database for plant image analysis software tools. Plant Methods 9, 38. 10.1186/1746-4811-9-38 24107223 PMC3853381

[B30] Martin W. N. Aggarwal J. K. (1983). Volumetric descriptions of objects from multiple views. IEEE Trans. Pattern Anal. Mach. Intell. 5, 150–158. 10.1109/TPAMI.1983.4767367 21869096

[B31] Mathworks (2015). Mathlab r2015b (Natick, MA). Accessed May 2016.

[B32] Minervini M. Abdelsamea M. M. Tsaftaris S. A. (2014). Image-based plant phenotyping with incremental learning and active contours. Ecol. Inf. 23, 35–48. 10.1016/j.ecoinf.2013.07.004

[B33] Minervini M. Scharr H. Tsaftaris S. A. (2015). Image analysis: the new bottleneck in plant phenotyping [applications corner]. Signal Process. Mag. IEEE 32, 126–131. 10.1109/MSP.2015.2405111

[B34] Moore C. R. Gronwall D. S. Miller N. D. Spalding E. P. (2013). Mapping quantitative trait loci affecting *Arabidopsis thaliana* seed morphology features extracted computationally from images. Genes Genomes Genet. 3, 109–118. 10.1534/g3.112.003806 23316443 PMC3538336

[B35] Müller-Linow M. Pinto-Espinosa F. Scharr H. Rascher U. (2015). The leaf angle distribution of natural plant populations: assessing the canopy with a novel software tool. Plant Methods 11, 11. 10.1186/s13007-015-0052-z 25774205 PMC4359433

[B36] Nagel K. Putz A. Gilmer F. Heinz K. Fischbach A. Pfeifer J. (2012). GROWSCREEN-Rhizo is a novel phenotyping robot enabling simultaneous measurements of root and shoot growth for plants grown in soil-filled rhizotrons. Funct. Plant Biol. 39, 891–904. 10.1071/FP12023 32480839

[B37] Next Instruments (2015). Seedcount. Accessed June 2015.

[B38] OpenGL.org (2015). Opengl Overview. Beaverton: The Khronos Group, Accessed June 2015.

[B39] Pape J.-M. Klukas C. (2015). 3-D histogram-based segmentation and leaf detection for rosette plants, in Computer Vision - ECCV 2014 Workshops, Vol. 8928 of Lecture Notes in Computer Science (Zurich: Springer International Publishing), 61–74.

[B40] Paproki A. Sirault X. Berry S. Furbank R. Fripp J. (2012). A novel mesh processing based technique for 3d plant analysis. BMC Plant Biol. 12:63. 10.1186/1471-2229-12-63 22553969 PMC3464618

[B41] Paulus S. Behmann J. Mahlein A.-K. Plümer L. Kuhlmann H. (2014). Low-cost 3d systems: Suitable tools for plant phenotyping. Sensors 14:3001. 10.3390/s140203001 24534920 PMC3958231

[B42] Potmesil M. (1987). Generating octree models of 3d objects from their silhouettes in a sequence of images. Comput. Vis. Graph. Image Process. 40, 1–29. 10.1016/0734-189X(87)90053-3

[B43] Pound M. P. French A. P. Fozard J. A. Murchie E. H. Pridmore T. P. (2016). A patch-based approach to 3d plant shoot phenotyping. Mach. Vis. Appl. [Epub ahead of print]. 10.1007/s00138-016-0756-8.

[B44] Pound M. P. French A. P. Murchie E. H. Pridmore T. P. (2014). Automated recovery of three-dimensional models of plant shoots from multiple color images. Plant Physiol. 166, 1688–1698. 10.1104/pp.114.248971 25332504 PMC4256878

[B45] Quan L. Tan P. Zeng G. Yuan L. Wang J. Kang S. B. (2006). Image-based plant modeling. ACM Trans. Graph. 25, 599–604. 10.1145/1141911.1141929

[B46] Regent Instruments (2000). Winseedle. Ville de Quèbec: Instruments Regent Inc., Accessed June 2015.

[B47] Roussel J. Fischbach A. Jahnke S. Scharr H. (2015). 3D surface reconstruction of plant seeds by volume carving, in Computer Vision Problems in Plant Phenotyping 2015 (Swansea).

[B48] Roussel J. Geiger F. Fischbach A. Jahnke S. Scharr H. (2016). Supplemental Material on “3D Surface Reconstruction of Plant Seeds by Volume Carving: Performance and Accuracies”. Available online at: http://www.fz-juelich.de/ibg/ibg-2/software 10.3389/fpls.2016.00745 PMC4895124 27375628

[B49] Santos T. T. Rodrigues G. C. (2015). Flexible three-dimensional modeling of plants using low- resolution cameras and visual odometry. Mach. Vis. Appl. [Epub ahead of print]. 10.1007/s00138-015-0729-3.

[B50] Silva L. O. L. A. Koga M. L. Cugnasca C. E. Costa A. H. R. (2013). Comparative assessment of feature selection and classification techniques for visual inspection of pot plant seedlings. Comput. Electron. Agricult. 97, 47–55. 10.1016/j.compag.2013.07.001

[B51] Sørensen T. (1948). A method of establishing groups of equal amplitude in plant sociology based on similarity of species content and its application to analyses of the vegetation on danish commons. Biol. Skr 5, 1–34.

[B52] Spalding E. P. Miller N. D. (2013). Image analysis is driving a renaissance in growth measurement. Curr. Opin. Plant Biol. 16, 100–104. 10.1016/j.pbi.2013.01.001 23352714

[B53] Szeliski R. (1993). Rapid octree construction from image sequences. CVGIP: Image Underst. 58, 23–32. 10.1006/ciun.1993.1029

[B54] Tanabata T. Shibaya T. Hori K. Ebana K. Yano M. (2012). Smartgrain: High-throughput phenotyping software for measuring seed shape through image analysis. Plant Physiol. 160, 1871–1880. 10.1104/pp.112.205120 23054566 PMC3510117

[B55] Topp C. N. Iyer-Pascuzzi A. S. Anderson J. T. Lee C.-R. Zurek P. R. Symonova O.. (2013). 3d phenotyping and quantitative trait locus mapping identify core regions of the rice genome controlling root architecture. Proc Natl. Acad. Sci. U.S.A. 110, E1695–E1704. 10.1073/pnas.1304354110 23580618 PMC3645568

[B56] Tsaftaris S. Noutsos C. (2009). Plant phenotyping with low cost digital cameras and image analytics, in Information Technologies in Environmental Engineering, eds Athanasiadis I. N. Rizzoli A. E. Mitkas P. A. Gómez J. M. (Berlin: Springer), 238–251.

[B57] van der Heijden G. Song Y. Horgan G. Polder G. Dieleman A. Bink M. (2012). SPICY: towards automated phenotyping of large pepper plants in the greenhouse. Funct. Plant Biol. 39, 870–877. 10.1071/FP12019 32480837

[B58] Wallenberg M. Felsberg M. Forssén P.-E. (2011). Leaf segmentation using the Kinect. in SSBA'11 Symposium on Image Analysis (Linköping), 1–4.

[B59] Wang L. Uilecan I. V. Assadi A. H. Kozmik C. A. Spalding E. P. (2009). HYPOTrace: image analysis software for measuring hypocotyl growth and shape demonstrated on Arabidopsis seedlings undergoing photomorphogenesis. Plant Physiol. 149, 1632–1637. 10.1104/pp.108.134072 19211697 PMC2663732

[B60] Weight C. Parnham D. Waites R. (2008). LeafAnalyser: a computational method for rapid and large-scale analyses of leaf shape variation. Plant J. 53, 578–586. 10.1111/j.1365-313X.2007.03330.x 18028263

[B61] Whan A. P. Smith A. B. Cavanagh C. R. Ral J.-P. F. Shaw L. M. Howitt C. A.. (2014). GrainScan: a low cost, fast method for grain size and colour measurements. Plant Methods 10, 1–10. 10.1186/1746-4811-10-23 25050131 PMC4105244

[B62] Yezzi A. J. Soatto S. (2003). Structure from motion for scenes without features, in Computer Vision and Pattern Recognition, 2003. Proceedings. *2003 IEEE Computer Society Conference on* (Madison), 525–532.

[B63] Zheng Y. Gu S. Edelsbrunner H. Tomasi C. Benfey P. (2011). Detailed reconstruction of 3d plant root shape, in Proceedings of the 2011 International Conference on Computer Vision, ICCV '11, (Washington, DC: IEEE Computer Society), 2026–2033.

